# Comparison of breast‐conserving surgery without radiotherapy and mastectomy in the treatment of elderly patients with early breast cancer: A PSM and SEER database study

**DOI:** 10.1002/cam4.6210

**Published:** 2023-06-03

**Authors:** Baiyang Fu, Xi Chen, Wenlong Liang, Yao Wang, Yuan Yao, Jianguo Zhang

**Affiliations:** ^1^ Department of Breast Surgery The Second Affiliated Hospital of Harbin Medical University Harbin China; ^2^ Department of Ultrasound The Second Affiliated Hospital of Harbin Medical University Harbin China

**Keywords:** breast cancer, breast‐conserving surgery (BCS), elderly, mastectomy, nomogram, propensity score matching (PSM), Surveillance, Epidemiology, and End Results (SEER)

## Abstract

**Background:**

At present, there is no research on which surgical method can lead to a better prognosis in elderly patients with early breast cancer. The purpose of this study was to establish a nomogram to predict the survival outcome of elderly patients with early breast cancer and to compare the prognosis of the breast‐conserving surgery (BCS) group who did not receive postoperative radiotherapy and the mastectomy group through risk stratification.

**Methods:**

This study included patients with early breast cancer aged ≥70 years from the Surveillance, Epidemiology, and End Results database (*n* = 20,520). The group was randomly divided into a development cohort (*n* = 14,363) and a validation cohort (*n* = 6157) according to a ratio of 7:3. Risk factors affecting overall survival (OS) and breast‐cancer‐specific survival (BCSS) were analyzed using univariate and multivariate Cox regression. Present results were obtained by constructing nomograms and risk stratifications. Nomograms were evaluated by the concordance index and calibration curve. Kaplan–Meier curves were established based on BCSS and analyzed using the log‐rank test.

**Results:**

Multivariate Cox regression results showed that age, race, pathological grade, T and N stages, and progesterone receptor (PR) status were independent risk factors for OS and BCSS in the BCS group and mastectomy group. Subsequently, they were incorporated into nomograms to predict 3‐ and 5‐year OS and BCSS in patients after BCS and mastectomy. The concordance index was between 0.704 and 0.832, and the nomograms also showed good calibration. The results of risk stratification showed that there was no survival difference between the BCS group and the mastectomy group in the low‐risk and high‐risk groups. In the middle‐risk group, BCS improved the BCSS of patients to a certain extent.

**Conclusion:**

This study constructed a well‐performing nomogram and risk stratification model to assess the survival benefit of BCS without postoperative radiotherapy in elderly patients with early breast cancer. The results of the study can help clinicians analyze the prognosis of patients and the benefits of surgical methods individually.

## 1 INTRODUCTION

Breast cancer is currently the most common malignancy and one of the leading causes of cancer death worldwide.^1^ Among them, elderly breast cancer patients aged 70 years or older accounted for more than 30% of newly diagnosed patients.^2^ Compared with younger patients, older breast cancer patients are characterized by an increased incidence of comorbidities, shorter life expectancy, and less aggressive tumors.^3^ However, most breast cancer clinical trials set an upper age limit of 70 years, resulting in a lack of Level 1 evidence for treatment of breast cancer in older adults.^4^, ^5^ Treatment plans are largely based on the extrapolation of clinical trial results in younger patients, which increases the risk of overtreatment in older patients.^6^

The strategy for treatment of breast cancer since the beginning of this century has shifted from a “maximum‐tolerated approach” to a “minimum‐effective approach”.^7^ Two randomized controlled trials, Cancer and Leukemia Group B (CALGB) 9343 and Prime II showed that adjuvant radiotherapy did not improve the overall survival (OS) benefit in elderly patients with hormone receptor (HR)‐positive early breast cancer after breast‐conserving surgery (BCS), providing a rationale for radiotherapy exemption.^8^, ^9^ Recent studies have shown that omission of radiotherapy after BCS has no effect on OS or breast‐cancer‐specific survival (BCSS) in older breast cancer patients with HR‐positive, human epidermal growth factor receptor 2 (HER2)‐positive, and triple‐negative breast cancer (TNBC) subtypes. Therefore, the omission of radiotherapy after BCS is safe in elderly patients with early breast cancer of all molecular subtypes.^10^, ^11^ The National Surgical Adjuvant Breast and Bowel Project (NSABP)‐B06 and the Milan trials compared the prognosis of BCS and mastectomy in early breast cancer. The follow‐up results at 20 years after surgery showed no significant difference in OS, making BCS the standard surgery for early breast cancer.^12^, ^13^ In recent years, several studies have shown that patients with early‐stage breast cancer who undergo BCS have higher survival rates than those who undergo mastectomy, further confirming the safety and efficacy of BCS.^14^, ^15^ However, these studies excluded older breast cancer patients. Therefore, it is necessary to design studies in elderly patients with early breast cancer to compare with mastectomy, to confirm the safety of BCS without radiotherapy and to improve the prognosis of patients in this age group.

This study was based on the Surveillance, Epidemiology, and End Results (SEER) database to compare the prognostic differences between BCS and mastectomy in elderly patients with early‐stage breast cancer who were exempt from postoperative radiotherapy. The safety of radiotherapy‐exempted BCS was determined by establishing a nomogram to predict prognosis and building a risk stratification model based on it.

## 2 METHODS

### 2.1 Patients

We searched the SEER database released in November 2020, covering approximately 30% of the US population from 18 registries.

**FIGURE 1:**
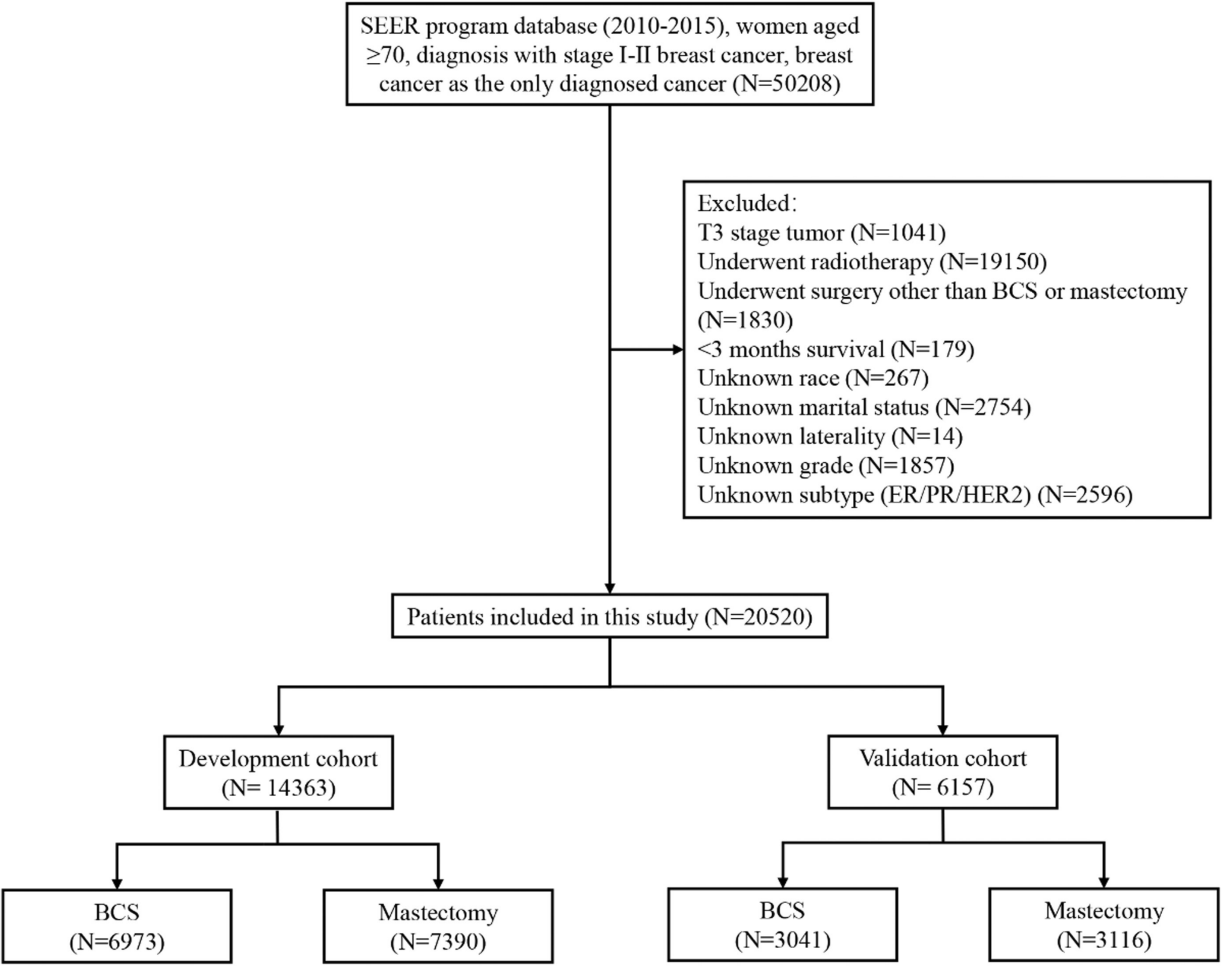
Patient selection flowchart. BCS, breast‐conserving surgery; ER, estrogen receptor; HER2, human epidermal growth factor receptor 2; PR, progesterone receptor; SEER, Surveillance, Epidemiology, and End Results.

Inclusion criteria: (1) The patient was diagnosed from 2010 to 2015 with Stage I‐II breast cancer; (2) female, aged ≥70 years; (3) breast cancer was the only primary malignant tumor; (4) the source of the report was neither an autopsy nor a death certificate. The exclusion criteria were as follows: (1) T3 stage tumor; (2) patients who underwent radiotherapy; (3) patients who underwent surgery other than BCS or mastectomy; (4) patients with <3 months of survival; and (5) patients with missing clinicopathological data. (Figure 1).

In this study, 20,520 patients were used to develop prognostic models and survival analysis, and BCSS and OS were considered outcome events. As the SEER database is public, informed consent and institutional research ethics committee approval are not needed.

### 2.2 Variables

In this study, 16 variables were selected: age at diagnosis, race, marital status, laterality, primary site, grade, histology, American Joint Committee on Cancer (AJCC) T stage, AJCC N stage, estrogen receptor (ER), progesterone receptor (PR), HER2 status, surgery type, chemotherapy, BCSS, and OS (Table 1).

**TABLE 1:** Baseline characteristics of patients.

Characteristics	Development cohort (*n* = 14,363)	Validation cohort (*n* = 6157)	*p*‐value
Age (years), *n* (%)			0.3977
70–74	4887 (34.02)	2102 (34.14)	
75–79	3747 (26.09)	1653 (26.85)	
80–84	2987 (20.80)	1284 (20.85)	
>85	2742 (19.09)	1118 (18.16)	
Race, *n* (%)			0.5498
White	12,020 (83.69)	5130 (83.32)	
Black	1187 (8.26)	508 (8.25)	
AIA	62 (0.43)	35 (0.57)	
API	1094 (7.62)	484 (7.86)	
Marital status, *n* (%)			0.8105
Unmarried	1288 (8.97)	545 (8.85)	
Married	13,075 (91.03)	5612 (91.15)	
Laterality, *n* (%)			0.9267
Left	7451 (51.88)	3189 (51.79)	
Right	6912 (48.12)	2968 (48.21)	
Primary site, *n* (%)			0.0525
Outer quadrant	5479 (38.15)	2451 (39.81)	
Inner quadrant	2898 (20.18)	1177 (19.12)	
Others	5986 (41.68)	2529 (41.08)	
Grade, *n* (%)			0.5822
I	4165 (29.00)	1823 (29.61)	
II	6785 (47.24)	2863 (46.50)	
III/IV	3413 (23.76)	1471 (23.89)	
Histology, *n* (%)			0.4261
Ductal	11,100 (77.28)	4745 (77.07)	
Lobular	1405 (9.78)	572 (9.29)	
Adenocarcinoma	766 (5.33)	343 (5.57)	
Other	1092 (7.60)	497 (8.07)	
AJCC T, *n* (%)			0.2315
T0/T1	9589 (66.76)	4164 (67.63)	
T2	4774 (33.24)	1993 (32.37)	
AJCC N, *n* (%)			0.1839
N0	12,072 (84.05)	5221 (84.80)	
N1	2291 (15.95)	936 (15.20)	
ER status, *n* (%)			0.9953
Positive	12,518 (87.15)	5367 (87.17)	
Negative	1845 (12.85)	790 (12.83)	
PR status, *n* (%)			0.2623
Positive	11,000 (76.59)	4670 (75.85)	
Negative	3363 (23.41)	1487 (24.15)	
HER2 status, *n* (%)			0.4412
Positive	1594 (11.10)	660 (10.72)	
Negative	12,769 (88.90)	5497 (89.28)	
Surgery type, *n* (%)			0.2751
BCS	6973 (48.55)	3041 (49.39)	
Mastectomy	7390 (51.45)	3116 (50.61)	
Chemotherapy, *n* (%)			0.5216
No/unknown	12,641 (88.01)	5439 (88.34)	
Yes	1722 (11.99)	718 (11.66)	
Follow‐up months, mean(SD)	66.619 (27.668)	66.764 (27.841)	0.7307

Abbreviations: AIA, American Indian/Alaska Native; AJCC, American Joint Committee on Cancer; API, Asian or Pacific Islander; BCS, breast‐conserving surgery; ER, estrogen receptor; HER2, human epidermal growth factor receptor 2; PR, progesterone receptor.

Age at diagnosis was regrouped into 70–74, 75–79, 80–84, and > 85 years. Race was divided into four groups: White, Black, American Indian/Alaska Native (AIA), Asian or Pacific Islander (API). Marital status was classified as unmarried, or married. Laterality was classified as left, or right. The primary site was divided into three categories: outer quadrant, inner quadrant, or other. The grade of tumor differentiation was divided into three categories: Grade I, Grade II, and Grade III/IV. Histology type was divided into four groups: ductal, lobular, adenocarcinoma, and other. The AJCC T stage was classified as T0/T1 and T2. The AJCC N stage was classified as N0 and N1. ER status was classified as positive or negative. PR status was classified as positive or negative. HER2 status was classified as positive or negative. Surgery type was classified as BCS or mastectomy. Chemotherapy is classified as no/unknown or yes, no/unknown includes the following three situations: (1) chemotherapy is not planned; (2) patients have contraindications to chemotherapy or patients refuse chemotherapy; (3) it is not clear whether it is recommended or used chemotherapy, as it was not stated in the patient's medical record. BCSS was defined as the time from the date of diagnosis to the date of death attributed to breast cancer. OS was defined as the length of time from diagnosis to death or the last follow‐up.

### 2.3 Statistical analysis

In our study, to guarantee the accuracy of the model, patients were randomized (7:3) into a development cohort and a validation cohort; categorical characteristics were compared using the Pearson chi‐square test, and continuous characteristics were compared using Student's *t*‐test. Before using the *t*‐test, it is necessary to use the Shapiro–Wilk test to determine whether the data are close to a normal distribution. To identify prognostic factors for BCS and mastectomy patients, univariate and multivariate Cox regression analyses were performed. The construction of prognostic nomograms according to the results of the multivariate analysis was evaluated from the aspects of discrimination and calibration. Discrimination was quantified by the concordance index (C‐index). The calibration was assessed against a calibration curve, which was plotted to analyze the correlation between predicted probabilities and actual results. To balance the baselines of the BCS and mastectomy groups, logistic regression was used for propensity score matching (PSM) with a caliper width of 0.01 and a ratio of 1:1 without replacement. In addition, the patients were divided into low‐risk, intermediate‐risk, and high‐risk groups based on the total score of the nomogram using X‐tile software. Kaplan–Meier curves were drawn and analyzed using the log‐rank test.

Statistical analyses were performed with R software (R Project for Statistical Computing, RRID:SCR_001905) version 4.1.0. Statistical significance was determined with a two‐sided *p* < 0.05.

## 3 RESULTS

### 3.1 Patient baseline characteristics

After a strict selection of inclusion and exclusion criteria, a total of 20,520 patients with early breast cancer were included in the study. They were randomly assigned to the development cohort (*n* = 14,363) and validation cohort (*n* = 6157) (Table 1). After the chi‐square test and *t*‐test analysis, there was no significant difference between the two cohorts, which proves that the verification value is high. Among them, more patients (51.2%, 10,506/20,520) underwent mastectomy, and fewer patients (48.8%, 10,014/20,520) underwent BCS under the premise of exempting radiotherapy. Differences in baseline characteristics between BCS and mastectomy patients in the development cohort are shown in Table 2. BCS patients were generally older, mostly white, with better pathological grades, mostly ductal type histologically, lower T and N stages, and mostly luminal type molecularly. At the same time, the proportion of chemotherapy in the BCS group was lower than that in the mastectomy group, which may be related to lower staging, better molecular typing, and comorbidities in elderly individuals.

**TABLE 2:** Baseline characteristics of patients in the development cohort.

Characteristics	BCS (*n* = 6973)	Mastectomy (*n* = 7390)	*p*‐value
Age (years), *n* (%)			<0.0001
70–74	2044 (29.31)	2843 (38.47)	
75–79	1717 (24.62)	2030 (27.47)	
80–84	1551 (22.24)	1436 (19.43)	
>85	1661 (23.82)	1081 (14.63)	
Race, *n* (%)			<0.0001
White	5986 (85.85)	6034 (81.65)	
Black	546 (7.83)	641 (8.67)	
AIA	22 (0.32)	40 (0.54)	
API	419 (6.01)	675 (9.13)	
Marital status, *n* (%)			0.2472
Unmarried	605 (8.68)	683 (9.24)	
Married	6368 (91.32)	6707 (90.76)	
Laterality, *n* (%)			0.7173
Left	3606 (51.71)	3845 (52.03)	
Right	3367 (48.29)	3545 (47.97)	
Primary site, *n* (%)			<0.0001
Outer quadrant	2841 (40.74)	2638 (35.70)	
Inner quadrant	1567 (22.47)	1331 (18.01)	
Others	2565 (36.78)	3421 (46.29)	
Grade, *n* (%)			<0.0001
I	2472 (35.45)	1693 (22.91)	
II	3202 (45.92)	3583 (48.48)	
III/IV	1299 (18.63)	2114 (28.61)	
Histology, *n* (%)			<0.0001
Ductal	5488 (78.70)	5612 (75.94)	
Lobular	563 (8.07)	842 (11.39)	
Adenocarcinoma	432 (6.20)	334 (4.52)	
Other	490 (7.03)	602 (8.15)	
AJCC T, *n* (%)			<0.0001
T0/T1	5455 (78.23)	4134 (55.94)	
T2	1518 (21.77)	3256 (44.06)	
AJCC N, *n* (%)			<0.0001
N0	6387 (91.60)	5685 (76.93)	
N1	586 (8.40)	1705 (23.07)	
ER status, *n* (%)			<0.0001
Positive	6346 (91.01)	6172 (83.52)	
Negative	627 (8.99)	1218 (16.48)	
PR status, *n* (%)			<0.0001
Positive	5645 (80.96)	5355 (72.46)	
Negative	1328 (19.04)	2035 (27.54)	
HER2 status, *n* (%)			<0.0001
Positive	621 (8.91)	973 (13.17)	
Negative	6352 (91.09)	6417 (86.83)	
Chemotherapy, *n* (%)			<0.0001
No/unknown	6372 (91.38)	6269 (84.83)	
Yes	601 (8.62)	1121 (15.17)	
Follow‐up months, mean (SD)	64.885 (27.060)	68.256 (28.133)	<0.0001

Abbreviations: AIA, American Indian/Alaska Native; AJCC, American Joint Committee on Cancer; API, Asian or Pacific Islander; BCS, breast‐conserving surgery; ER, estrogen receptor; HER2, human epidermal growth factor receptor 2; PR, progesterone receptor.

### 3.2 Cox regression identifies independent prognostic factors

To develop the prognostic model, we used Cox regression models to screen for prognostic factors affecting OS and BCSS of BCS and mastectomy. First, all 14 original variables were included in the regression model (Table 3). Univariate Cox regression found that six variables were risk factors for OS and BCSS in patients with BCS and mastectomy: age at diagnosis, grade, T stage, ER status, PR status, and chemotherapy. In addition, race was a risk factor for OS, and histology, N stage, and HER2 status were risk factors for BCSS in the BCS group. In the mastectomy group, race, primary site, and N stage were risk factors for OS, and race, marital status, primary site, histology, N stage, and HER2 status were risk factors for BCSS. Subsequently, variables with statistical significance (*p* < 0.05) in univariate analysis were included in multivariate analysis (Table 4). We defined OS in the BCS group, BCSS in the BCS group, OS in the mastectomy group, and BCSS in the mastectomy group as four prognostic cohorts. When a variable screened by multivariate analysis was associated with prognosis in at least three cohorts, it was considered a prognostic factor that could be included in the nomogram analysis. Multivariate Cox regression found that three variables (age, grade, and T stage) were associated with prognosis in all four cohorts. In addition, three variables (race, N stage, PR status) were associated with prognosis in three of all four cohorts.

**TABLE 3:** Univariate Cox regression analysis of prognostic factors for patients.

Characteristics	OS				BCSS			
	BCS		Mastectomy		BCS		Mastectomy	
	HR [95% CI]	*p*‐value	HR [95% CI]	*p*‐value	HR [95% CI]	*p*‐value	HR [95% CI]	*p*‐value
Age (years), *n* (%)								
70–74	Reference		Reference		Reference		Reference	
75–79	1.73 [1.49–2.01]	<0.001	1.62 [1.43–1.83]	<0.001	1.09 [0.8–1.47]	0.589	1.2 [0.95–1.52]	0.124
80–84	2.79 [2.42–3.21]	<0.001	3.06 [2.72–3.44]	<0.001	1.31 [0.97–1.78]	0.075	1.87 [1.48–2.36]	<0.001
>85	5.8 [5.09–6.6]	<0.001	5.17 [4.6–5.81]	<0.001	2.59 [1.99–3.38]	<0.001	3.05 [2.42–3.84]	<0.001
Race, *n* (%)								
White	Reference		Reference		Reference		Reference	
Black	1.09 [0.94–1.26]	0.264	0.99 [0.86–1.14]	0.866	1.31 [0.94–1.84]	0.113	1.18 [0.9–1.56]	0.227
AIA	1.23 [0.64–2.36]	0.538	1.08 [0.64–1.83]	0.771	1.65 [0.41–6.65]	0.478	0.99 [0.32–3.09]	0.992
API	0.69 [0.57–0.85]	<0.001	0.63 [0.53–0.75]	<0.001	1.1 [0.74–1.64]	0.638	0.59 [0.41–0.85]	0.005
Marital status, *n* (%)								
Unmarried	Reference		Reference		Reference		Reference	
Married	1.04 [0.9–1.21]	0.589	0.99 [0.86–1.14]	0.931	0.93 [0.66–1.32]	0.7	1.52 [1.08–2.15]	0.018
Laterality, *n* (%)								
Left	Reference		Reference		Reference		Reference	
Right	1.04 [0.96–1.13]	0.385	0.98 [0.91–1.07]	0.686	1.03 [0.84–1.25]	0.799	1.02 [0.86–1.2]	0.832
Primary site, *n* (%)								
Outer quadrant	Reference		Reference		Reference		Reference	
Inner quadrant	0.94 [0.84–1.05]	0.273	0.97 [0.86–1.09]	0.591	0.98 [0.76–1.27]	0.878	0.97 [0.75–1.25]	0.788
Others	1.06 [0.96–1.16]	0.243	1.1 [1–1.2]	0.039	1.01 [0.81–1.27]	0.915	1.23 [1.02–1.48]	0.032
Grade, *n* (%)								
I	Reference		Reference		Reference		Reference	
II	1.21 [1.1–1.33]	<0.001	1.19 [1.06–1.32]	0.002	2.37 [1.71–3.29]	<0.001	2.27 [1.65–3.11]	<0.001
III/IV	1.75 [1.56–1.96]	<0.001	1.63 [1.451.82]	<0.001	8.9 [6.49–12.19]	<0.001	5.55 [4.07–7.58]	<0.001
Histology, *n* (%)								
Ductal	Reference		Reference		Reference		Reference	
Lobular	0.98 [0.84–1.15]	0.841	0.89 [0.78–1.02]	0.085	0.88 [0.6–1.29]	0.512	0.7 [0.52–0.95]	0.021
Adenocarcinoma	1.01 [0.86–1.2]	0.882	0.96 [0.79–1.17]	0.697	0.38 [0.2–0.72]	0.003	0.26 [0.12–0.56]	<0.001
Other	0.93 [0.79–1.09]	0.373	0.97 [0.83–1.13]	0.677	1 [0.68–1.46]	0.99	1.08 [0.81–1.45]	0.596
AJCC T, *n* (%)								
T0/T1	Reference		Reference		Reference		Reference	
T2	2.28 [2.09–2.48]	<0.001	1.78 [1.64–1.93]	<0.001	5.33 [4.36–6.51]	<0.001	3.29 [2.74–3.95]	<0.001
AJCC N, *n* (%)								
N0	Reference		Reference		Reference		Reference	
N1	1.13 [0.98–1.3]	0.085	1.46 [1.34–1.6]	<0.001	2.91 [2.28–3.72]	<0.001	2.57 [2.16–3.04]	<0.001
ER status, *n* (%)								
Positive	Reference		Reference		Reference		Reference	
Negative	1.39 [1.22–1.58]	<0.001	1.48 [1.34–1.63]	<0.001	4.09 [3.27–5.11]	<0.001	2.72 [2.27–3.25]	<0.001
PR status, *n* (%)								
Positive	Reference		Reference		Reference		Reference	
Negative	1.28 [1.16–1.41]	<0.001	1.43 [1.31–1.56]	<0.001	3.47 [2.84–4.24]	<0.001	2.71 [2.29–3.2]	<0.001
HER2 status, *n* (%)								
Positive	Reference		Reference		Reference		Reference	
Negative	0.9 [0.78–1.03]	0.125	1 [0.88–1.13]	0.962	0.43 [0.33–0.55]	<0.001	0.73 [0.58–0.91]	0.005
Chemotherapy, *n* (%)								
No/unknown	Reference		Reference		Reference		Reference	
Yes	0.71 [0.6–0.84]	<0.001	0.64 [0.56–0.73]	<0.001	2.6 [2.03–3.33]	<0.001	1.32 [1.07–1.63]	0.009

Abbreviations: AIA, American Indian/Alaska Native; AJCC, American Joint Committee on Cancer; API, Asian or Pacific Islander; BCS, breast‐conserving surgery; BCSS, breast‐cancer‐specific survival; CI, confidence interval; ER, estrogen receptor; HER2, human epidermal growth factor receptor 2; HR, hazard ratio; OS, overall survival; PR, progesterone receptor.

**TABLE 4:** Multivariate Cox regression analysis of prognostic factors for patients.

Characteristics	OS				BCSS			
	BCS		Mastectomy		BCS		Mastectomy	
	HR [95% CI]	*p*‐value	HR [95% CI]	*p*‐value	HR [95% CI]	*p*‐value	HR [95% CI]	*p*‐value
Age (years), *n* (%)								
70–74	Reference		Reference		Reference		Reference	
75–79	1.78 [1.53–2.06]	<0.001	1.55 [1.37–1.75]	<0.001	1.22 [0.9–1.66]	0.197	1.15 [0.91–1.46]	0.239
80–84	2.86 [2.48–3.31]	<0.001	2.72 [2.41–3.07]	<0.001	1.7 [1.24–2.33]	0.001	1.58 [1.24–2.01]	<0.001
>85	5.56 [4.85–6.36]	<0.001	4.3 [3.8–4.86]	<0.001	2.9 [2.17–3.87]	<0.001	2.22 [1.74–2.85]	<0.001
Race, *n* (%)								
White	Reference		Reference		‐	‐	Reference	
Black	1.26 [1.08–1.46]	0.003	0.96 [0.83–1.11]	0.592	‐	‐	1.04 [0.79–1.37]	0.778
AIA	1.56 [0.81–3.01]	0.182	1.29 [0.76–2.18]	0.349	‐	‐	1.08 [0.35–3.38]	0.891
API	0.73 [0.6–0.89]	0.002	0.65 [0.55–0.77]	<0.001	‐	‐	0.6 [0.42–0.87]	0.006
Marital status, *n* (%)								
Unmarried	‐	‐	‐	‐	‐	‐	Reference	
Married	‐	‐	‐	‐	‐	‐	1.43 [1.01–2.03]	0.044
Primary site, *n* (%)								
Outer quadrant	‐	‐	Reference		‐	‐	Reference	
Inner quadrant	‐	‐	0.98 [0.87–1.11]	0.747	‐	‐	1.05 [0.81–1.35]	0.737
Others	‐	‐	1.04 [0.95–1.14]	0.386	‐	‐	1.22 [1.01–1.47]	0.04
Grade, *n* (%)								
I	Reference		Reference		Reference		Reference	
II	1.08 [0.98–1.19]	0.142	1.07 [0.96–1.19]	0.245	1.7 [1.21–2.37]	0.002	1.74 [1.26–2.39]	0.001
III/IV	1.37 [1.2–1.55]	<0.001	1.27 [1.12–1.45]	<0.001	3.63 [2.53–5.21]	<0.001	2.95 [2.1–4.14]	<0.001
Histology, *n* (%)								
Ductal	‐	‐	‐	‐	Reference		Reference	
Lobular	‐	‐	‐	‐	1.12 [0.76–1.65]	0.576	0.9 [0.66–1.23]	0.508
Adenocarcinoma	‐	‐	‐	‐	0.57 [0.3–1.08]	0.087	0.44 [0.21–0.92]	0.03
Other	‐	‐	‐	‐	1.06 [0.72–1.56]	0.757	1.07 [0.8–1.44]	0.628
AJCC T, *n* (%)								
T0/T1	Reference		Reference		Reference		Reference	
T2	1.87 [1.7–2.05]	<0.001	1.43 [1.31–1.56]	<0.001	2.83 [2.27–3.53]	<0.001	2.27 [1.88–2.75]	<0.001
AJCC N, *n* (%)								
N0	‐	‐	Reference		Reference		Reference	
N1	‐	‐	1.37 [1.25–1.51]	<0.001	1.54 [1.19–2]	0.001	2.12 [1.77–2.53]	<0.001
ER status, *n* (%)								
Positive	Reference		Reference		Reference		Reference	
Negative	1.17 [0.98–1.39]	0.081	1.18 [1.03–1.36]	0.019	1.25 [0.92–1.7]	0.155	1.26 [0.98–1.62]	0.067
PR status, *n* (%)								
Positive	Reference		Reference		Reference		Reference	
Negative	1.05 [0.93–1.19]	0.432	1.25 [1.11–1.4]	<0.001	1.64 [1.26–2.15]	<0.001	1.9 [1.52–2.39]	<0.001
HER2 status, *n* (%)								
Positive	‐	‐	‐	‐	Reference		Reference	
Negative	‐	‐	‐	‐	0.94 [0.71–1.24]	0.68	1.26 [0.99–1.59]	0.059
Chemotherapy, *n* (%)								
No/unknown	Reference		Reference		Reference		Reference	
Yes	0.87 [0.72–1.05]	0.143	0.66 [0.57–0.76]	<0.001	1.17 [0.86–1.59]	0.309	0.72 [0.57–0.92]	0.008

Abbreviations: AIA, American Indian/Alaska Native; AJCC, American Joint Committee on Cancer; API, Asian or Pacific Islander; BCS, breast‐conserving surgery; CI, confidence interval; ER, estrogen receptor; HER2, human epidermal growth factor receptor 2; HR, hazard ratio; OS, overall survival; BCSS, breast‐cancer‐specific survival; PR, progesterone receptor.

### 3.3 Construction and validation of the nomogram for predicting BCSS and OS in BCS and mastectomy patients

**FIGURE 2:**
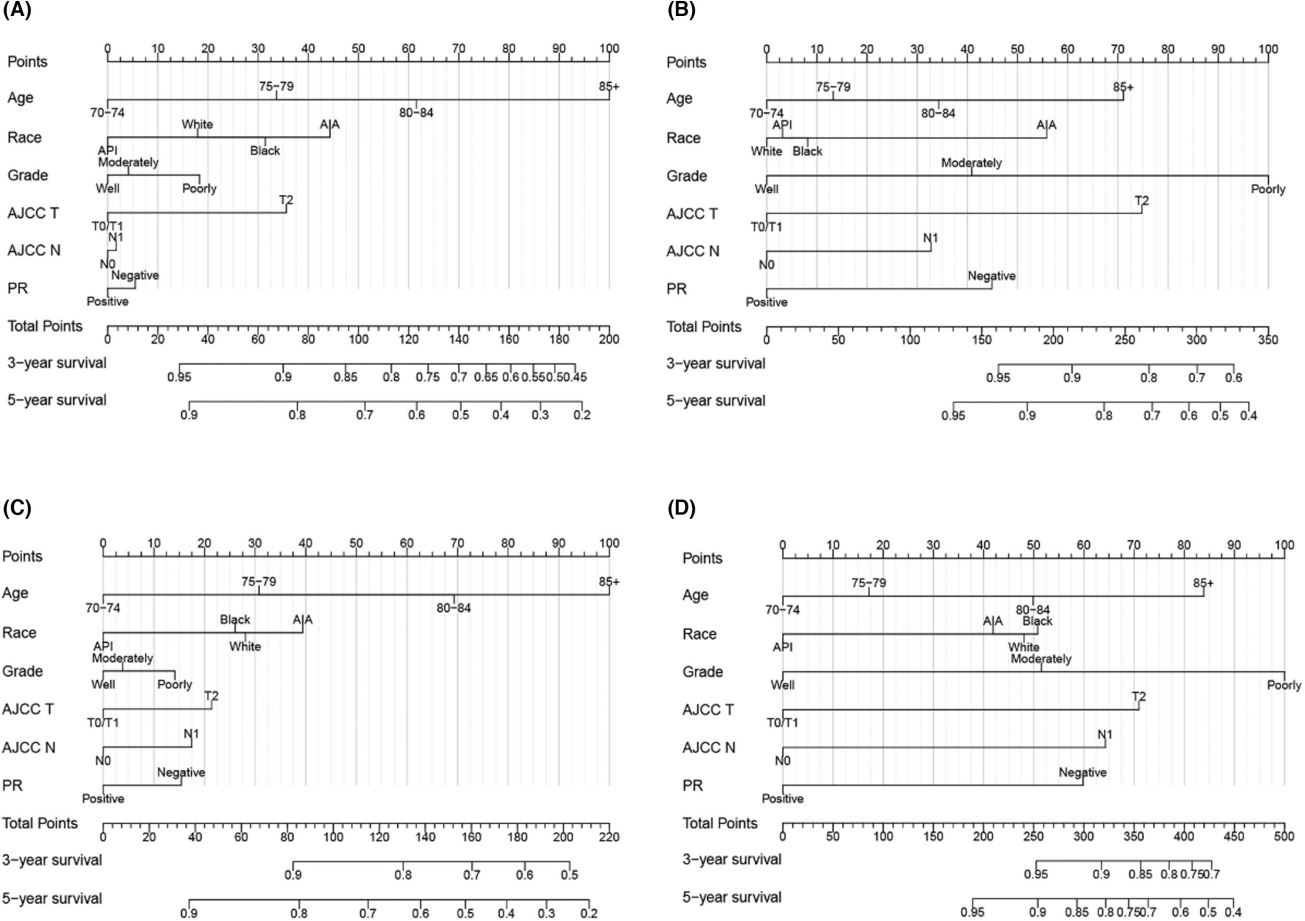
Nomogram for predicting OS and BCSS elderly patients with early breast cancer. (A) OS for patients with BCS. (B) BCSS for patients with BCS. (C) OS for patients with mastectomy. (D) BCSS for patients with mastectomy. AIA, American Indian/Alaska Native; API, Asian or Pacific Islander; BCS, breast‐conserving surgery; PR, progesterone receptor.

Based on the 6 prognostic factors (age, race, grade, T stage, N stage, and PR status) screened out by multivariate Cox regression, nomograms of OS and BCSS for predicting BCS and mastectomy at 3 and 5 years were established (Figure 2). The patient's prognosis can be predicted by adding the corresponding scores of the patients' clinical variables (Table 5) to obtain the total score. The higher the total score, the worse the patient's prognosis. On the whole, patients with younger ages, lower pathological grades, lower T and N stages, and positive PR status had a better prognosis.

**TABLE 5:** Scores of clinical characteristics in each nomogram.

Characteristics	OS		BCSS	
	BCS	Mastectomy	BCS	Mastectomy
Age (years), *n* (%)				
70–74	0	0	0	0
75–79	34	31	13	17
80–84	62	69	34	50
>85	100	100	71	84
Race, *n* (%)				
White	18	28	0	48
Black	31	26	8	51
AIA	44	39	56	42
API	0	0	3	0
Grade, *n* (%)				
I	0	0	0	0
II	4	4	41	52
III/IV	18	14	100	100
AJCC T, *n* (%)				
T0/T1	0	0	0	0
T2	36	21	75	71
AJCC N, *n* (%)				
N0	0	0	0	0
N1	2	17	33	64
PR status, *n* (%)				
Positive	0	0	0	0
Negative	5	15	45	60

Abbreviations: AIA, American Indian/Alaska Native; AJCC, American Joint Committee on Cancer; API, Asian or Pacific Islander; BCS, breast‐conserving surgery; BCSS, breast‐cancer‐specific survival; OS, overall survival; PR, progesterone receptor.

**FIGURE 3:**
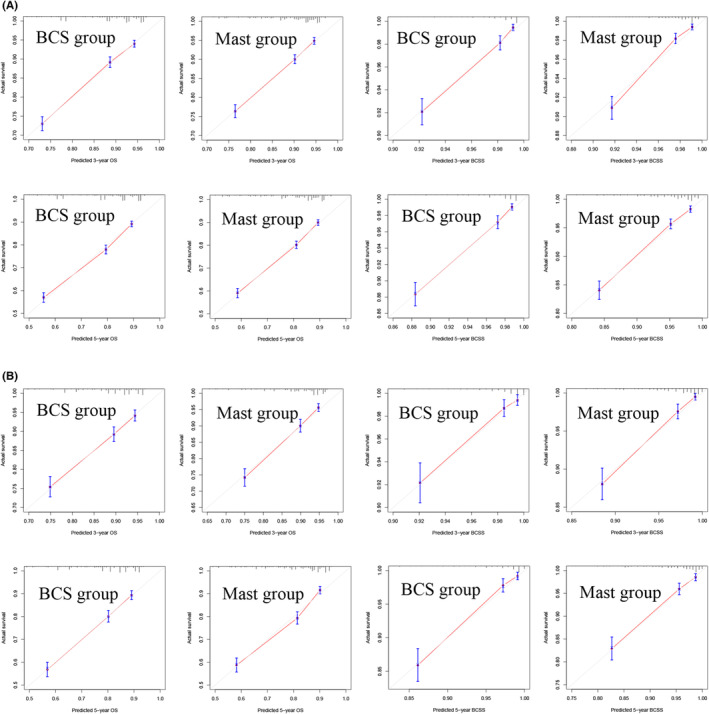
Calibration curves in the development cohort (A) and validation cohort (B). BCS, breast‐conserving surgery; Mast, mastectomy.

We used the development cohort and the validation cohort to verify the discrimination and calibration of the model. The C‐indices of the four nomograms ranged from 0.714 to 0.829 in the development cohort and 0.704 to 0.832 in the validation cohort (Table 6). Calibration curves of survival rates at 3 and 5 years in the BCS and mastectomy groups showed satisfactory internal model‐fitting capabilities (Figure 3). Excellent discrimination and calibration demonstrated the accuracy of the prognostic model.

**TABLE 6:** C‐index for development and validation cohort.

Model	Development cohort (95% CI)	Validation cohort (95% CI)
OS for patients with BCS	0.729 (0.711–0.746)	0.704 (0.676–0.733)
BCSS for patients with BCS	0.829 (0.801–0.857)	0.831 (0.782–0.880)
OS for patients with Mastectomy	0.714 (0.696–0.732)	0.733 (0.706–0.760)
BCSS for patients with Mastectomy	0.815 (0.790–0.840)	0.832 (0.799–0.864)

Abbreviations: BCS, breast‐conserving surgery; BCSS, breast‐cancer‐specific survival; CI, confidence interval; C‐index, concordance index; OS, overall survival.

### 3.4 Establishment of patient risk stratification after PSM

**FIGURE 4:**
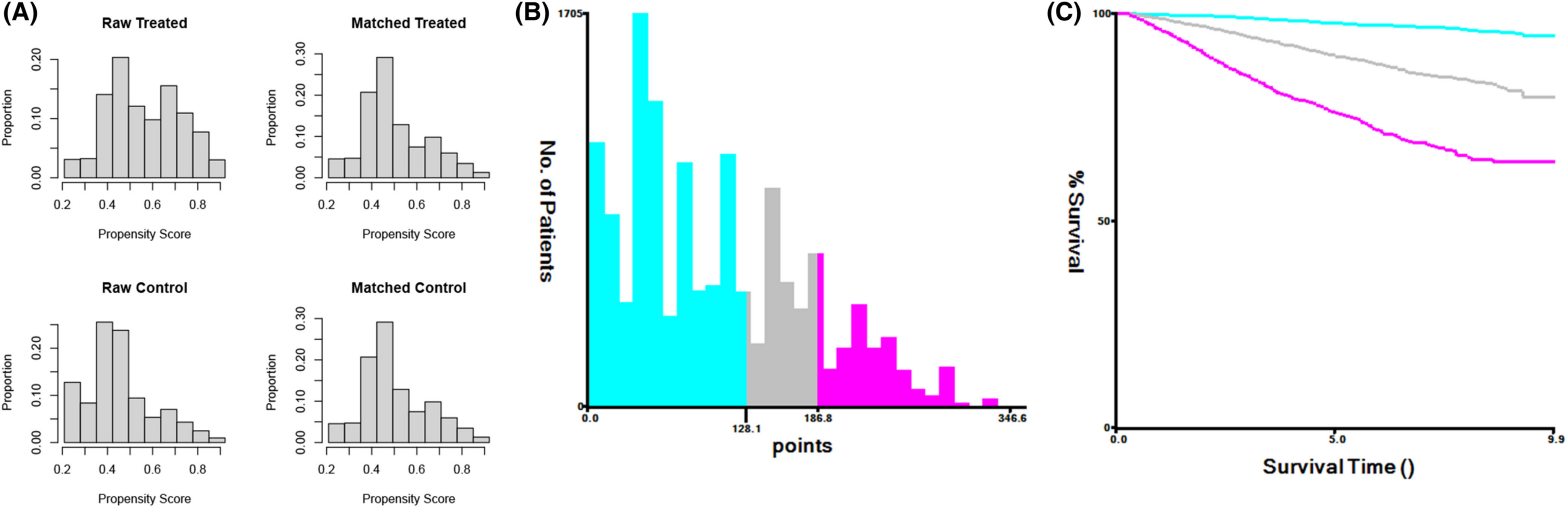
Risk stratification data analysis. (A) The distribution of propensity scores for matched and unmatched patients. (B, C) X‐tile calculates cut‐off values for scores in elderly breast cancer patients.

**FIGURE 5:**
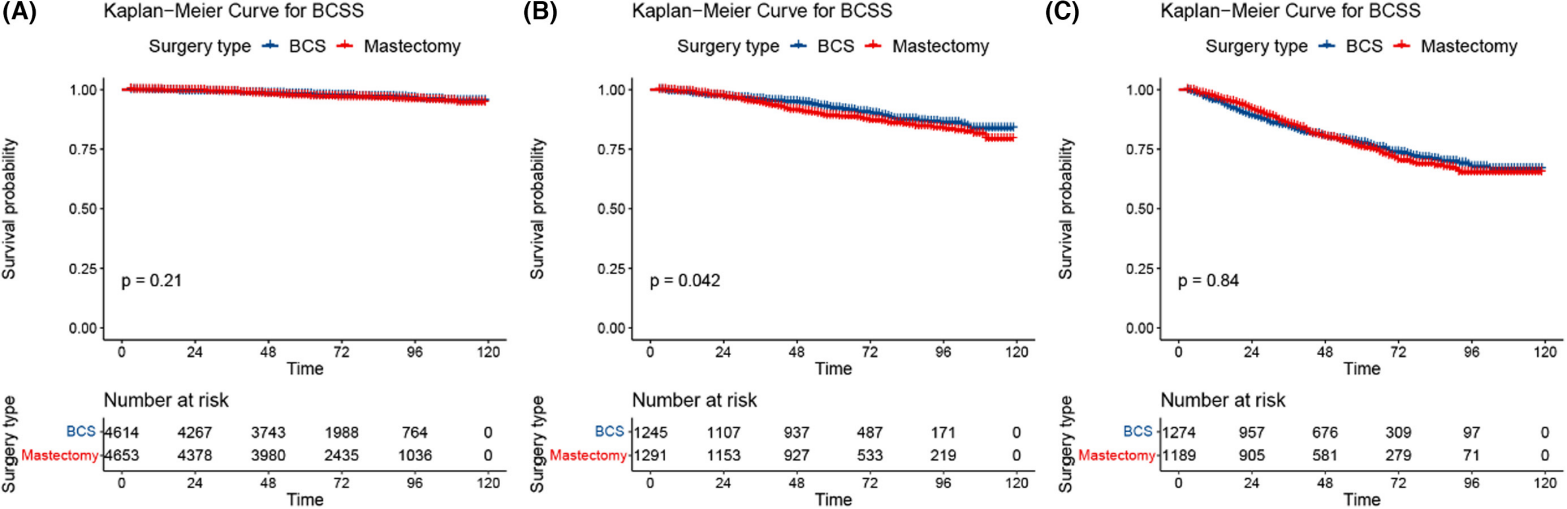
Survival benefit of BCS in the risk stratification groups. (A) BCSS in the low‐risk group. (B) BCSS in the intermediate‐risk group. (C) BCSS in the high‐risk group. BCS: breast‐conserving surgery.

In elderly patients, there are more confounding factors affecting OS, so BCSS is a better indicator of treatment benefit. Therefore, we established a risk stratification of patients with BCSS based on a model that predicted BCSS in patients in the BCS group. Furthermore, since this was a retrospective study with selection bias, PSM was used to make the balance of clinical variables comparable between patients in the BCS group and those in the mastectomy group. In addition, the development cohort and validation cohort were combined into a new ensemble for analysis, and logistic regression was used for PSM. After matching the 6 factors of age, race, grade, T stage, N stage, and PR status, 14,266 patients were included, and there were 7133 patients in the BCS group and the mastectomy group (Figure 4A). Patients were divided into low‐risk, intermediate‐risk, and high‐risk prognostic groups according to each patient's nomogram score. X‐tile software was used to calculate the cutoff value of the total score of patients: low‐risk group (9267/14,266, 65.0%, total score < 128), intermediate‐risk group (2536/14,266, 17.8%, total score 128–186), high‐risk group (2463/14,266, 17.2%, total score > 186) (Figure 4B,C). To avoid increasing Type I errors, we split the total data set into three independent data sets according to the cutoff value for statistical analysis. There was no significant difference in BCSS between the BCS group and the mastectomy group in the low‐risk and high‐risk groups (*p* = 0.21, *p* = 0.84). In the intermediate‐risk group, BCS improved BCSS to some extent (*p* = 0.042) (Figure 5).

## 4 DISCUSSION

For elderly patients with early‐stage breast cancer, the survival outcomes of radiotherapy‐omitted BCS and mastectomy are a question worthy of discussion. Studies have shown that the total number of comorbidities, such as hypertension, heart disease, chronic obstructive pulmonary disease, diabetes, and previous malignant tumors, increases with age, and these concomitant diseases also increase the risk of death in elderly breast cancer patients.^16^ At the same time, with increasing age, the proportion of breast cancer deaths attributable to breast cancer in elderly breast cancer patients gradually decreased, while cardiovascular and cerebrovascular diseases and other concomitant diseases became increasingly important as the cause of death.^16^, ^17^ Therefore, concomitant diseases should receive as much attention as cancer itself when studying the prognosis of older breast cancer patients. Previous studies have found that postoperative radiation damage to various structures and tissues in the heart can lead to a series of radiation‐induced cardiovascular diseases. There was a statistically significant difference in cardiovascular mortality between patients who received and did not receive postoperative radiotherapy.^18^ Meanwhile, elderly patients who underwent mastectomy had a higher risk of postoperative complications than patients who underwent BCS. Due to the longer surgical incision and larger surgical scope, patients undergoing mastectomy will have more surgical complications, including incision infection, incision dehiscence, bleeding, pain, subcutaneous hematoma, ischemic necrosis of skin flap, and fat liquefaction. In addition, greater trauma also brings more systemic complications, including anemia, cardiovascular and cerebrovascular complications, thromboembolism, etc.^19^ Compared with mastectomy, BCS has obvious advantages in reducing psychological morbidity, reducing anxiety, and improving body image and self‐esteem.^20^, ^21^ Is there a group of elderly patients who can avoid radiation therapy and mastectomy without sacrificing survival? This study included 20,520 elderly patients with early breast cancer aged 70 years or older. By establishing a prognostic model and risk stratification, it was found that the BCS group and the mastectomy group had the same BCSS in the low‐risk group and high‐risk group. Even in the intermediate‐risk group, the prognosis of the BCS group omitting radiotherapy was better than that of the mastectomy group. The prognosis depends primarily on risk stratification. This means that elderly patients with early breast cancer can avoid the complications of radiotherapy and mastectomy without sacrificing survival. We believe that these nomograms and risk stratification can help clinicians accurately determine the delicate treatment balance between increased breast cancer survival and potential toxicity while reducing the incidence of overtreatment and undertreatment.

Given the potential complications of postoperative radiotherapy in elderly breast cancer patients, researchers have been exploring the possibility of omitting postoperative radiotherapy after BCS. CALGB 9343 included 636 ER‐positive elderly patients with early breast cancer aged ≥70 years. After a median follow‐up of 12.6 years, it was confirmed that radiotherapy after BCS can reduce the risk of local recurrence, but this cannot be translated into OS and BCSS being beneficial.^8^ Prime II included 1326 HR‐positive elderly patients with early breast cancer aged ≥65 years. After a median follow‐up of 5 years, it was confirmed that radiotherapy after BCS could reduce the risk of local recurrence, but it could not improve OS. The recurrence rate in the ipsilateral breast was low enough for the patient to consider not undergoing radiation therapy.^9^ Two retrospective studies also found that the rate of local recurrence after BCS decreased with age, regardless of radiotherapy. The omission of radiation therapy is reasonable for patients with limited life expectancy or those whose radiation therapy may have serious consequences.^22^, ^23^ A meta‐analysis of 10,801 breast cancer patients in 17 randomized trials found that radiotherapy after BCS reduced local recurrence and reduced the risk of long‐term mortality. However, this is a study of people of all ages. In this study, the elderly over 70 years old accounted for only 19% (2056/10,801) of the included patients.^24^ Compared with the study of whether postoperative radiotherapy can be waived, there are relatively few studies comparing the prognosis of BCS and mastectomy with postoperative radiotherapy omitted. A randomized controlled trial in the UK included 222 elderly breast cancer patients over 70 years old. The study showed that patients who chose BCS were older, but there was no significant difference in OS between the BCS group and the mastectomy group.^25^ A retrospective study based on the SEER database included 1784 elderly patients with early breast cancer aged ≥70 years. It was found that compared with the mastectomy group, the BCS group omitting postoperative radiotherapy had better OS and BCSS.^3^ Although most older adults with early‐stage breast cancer are candidates for BCS, available data suggest that older patients with T1‐T2 stages undergo mastectomy more frequently than younger patients.^26^ Therefore, it is necessary to construct specific screening tools to provide the most appropriate treatment options for elderly patients.

Our study constructed a prognostic model and performed risk stratification analysis on elderly patients with early breast cancer, providing clinicians with an auxiliary treatment decision‐making tool to improve the prognosis of such patients. After Cox regression analysis, we concluded that age, race, pathological grade, T and N stages, and PR status were independent prognostic factors, and based on this, four prognostic models were constructed to predict OS and BCSS. Risk stratification was subsequently established based on the BCSS model predicting BCS patients. The low‐risk group with a score of less than 128 has the characteristics of younger age, higher possibility of the White race, better pathological grade, lower T and N stages, and positive PR. Patients in the low‐risk group accounted for more than half (65.0%) of all patients. These patients can obtain the same prognosis regardless of whether they choose BCS or mastectomy, so they can safely choose BCS that omits radiotherapy. Patients in the high‐risk group with a score higher than 186 were older, more likely to be American Indian/Alaska Native race, had worse pathological grades, had higher T and N stages, and had negative PR. Compared with the low‐risk group, the prognosis of the high‐risk group was worse, but the choice of BCS and mastectomy did not lead to a difference in survival, so the corresponding surgical method can be selected according to the actual situation. The characteristics of the intermediate‐risk group with a score of 128–186 are between those of the low‐risk group and the high‐risk group. The prognosis of this group of patients who choose BCS is slightly better than that of mastectomy, so this group of patients has more reasons to choose BCS that omits radiotherapy. As an example, an 82‐year‐old woman of AIA ethnicity was diagnosed with T1N0M0 breast cancer. The biopsy results showed that the pathological grade was grade II, and the molecular type was PR negative. According to our nomogram prediction, no matter what kind of surgery she received, her 3‐year and 5‐year OS were over 75% and 60%, respectively, and her 3‐year and 5‐year BCSS were over 93% and 90%, respectively. Given that she was classified as an intermediate‐risk patient with a score of 176 in risk stratification, choosing BCS could lead to a better prognosis.

To the best of our knowledge, this study is the first to establish a nomogram based on a large amount of data to evaluate the benefit of surgery in elderly patients with early breast cancer, but this study still has some limitations. First, we may have overlooked potential prognostic factors because details of adjuvant therapy and comorbidities in older adults were not reported in the SEER database. Second, this study lacks validation from external data, and we will use our data to validate these predictive models in further studies. Last but not least, although both CALGB 9343 and Prime II pointed out that the reduction in the local recurrence rate did not translate into a survival benefit, there is a lack of progression‐free survival‐related data in the SEER database, and we have no way of knowing whether the local recurrence itself or the relevant treatment after recurrence affect the survival of the elderly. Therefore, whether recurrence after BCS in the elderly exempted from radiotherapy affects survival needs to be verified by prospective studies.

## 5 CONCLUSION

This study analyzed the survival differences caused by surgical methods in elderly patients with early breast cancer by constructing a prognostic model and risk stratification. In the low‐risk and high‐risk groups, the BCS group omitting postoperative radiotherapy had the same prognosis as the mastectomy group. In the intermediate‐risk group, the prognosis of the BCS group omitting postoperative radiotherapy was better than that of the mastectomy group. This study can help clinicians analyze the prognosis of patients and the benefits of surgical methods individually. We hope that future prospective studies will confirm our conclusions.

## AUTHOR CONTRIBUTIONS

**Baiyang Fu:** Conceptualization (lead); data curation (lead); formal analysis (lead); investigation (lead); methodology (lead); resources (equal); software (lead); validation (lead); visualization (lead); writing – original draft (lead); writing – review and editing (equal). **Xi Chen:** Conceptualization (equal); formal analysis (equal); investigation (equal); methodology (equal); project administration (lead); writing – review and editing (lead). **Wenlong Liang:** Conceptualization (equal); data curation (equal); methodology (equal); project administration (equal); validation (equal); visualization (equal); writing – review and editing (equal). **Yao Wang:** Conceptualization (equal); formal analysis (equal); methodology (lead); visualization (equal); writing – review and editing (equal). **Yuan Yao:** Conceptualization (equal); data curation (equal); methodology (lead); software (equal); validation (equal); visualization (lead); writing – review and editing (equal). **Jianguo Zhang:** Conceptualization (lead); data curation (equal); funding acquisition (lead); project administration (lead); resources (lead); supervision (lead); writing – review and editing (lead).

## FUNDING INFORMATION

There is no funding to report.

## CONFLICT OF INTEREST STATEMENT

The authors declare that there is no conflict of interest regarding the publication of this article.

## Data Availability

The dataset for this study can be obtained from the SEER database (https://seer.cancer.gov/).
